# The Relation between Parameters of Physical Performance and Depression in Consecutive Hospitalized Geriatric Patients with Heart Failure

**DOI:** 10.3390/nu16193392

**Published:** 2024-10-06

**Authors:** Malgorzata Kupisz-Urbańska, Urszula Religioni, Wiktoria Niegowska, Julia Szydlik, Piotr Czapski, Siamala Sinnadurai, Katarzyna Januszewska, Ada Sawicka, Agnieszka Drab, Jarosław Pinkas, Piotr Jankowski

**Affiliations:** 1 Department of Geriatrics, Medical Centre of Postgraduate Education, 00-416 Warsaw, Poland; 2 Department of Internal Medicine and Geriatric Cardiology, Medical Centre of Postgraduate Education, 00-416 Warsaw, Poland; wniegowska@gmail.com (W.N.); jszydlik@gmail.com (J.S.); piotr4321@gmail.com (P.C.); kjanuszewska@gmail.com (K.J.); asaw@wp.pl (A.S.); piotrjankowski.eu@gmail.com (P.J.); 3 Department of Lifestyle Medicine, School of Public Health, Centre of Postgraduate Medical Education, 01-826 Warsaw, Poland; urszula.religioni@cmkp.edu.pl (U.R.); jpinkas@cmkp.edu.pl (J.P.); 4 Department of Epidemiology and Health Promotion, School of Public Health, Centre of Postgraduate Medical Education, 00-416 Warsaw, Poland; sinnadurais@gmail.com; 5 Department of Medical Informatics and Statistics with E-Learning Laboratory, Medical University of Lublin, 20-090 Lublin, Poland; agnieska.drab@gmail.com

**Keywords:** depression, Five Times Sit to Stand Test, Timed Up and Go Test, heart failure, comorbidity, Geriatric Depression Scale, physical activity, cardiovascular risk

## Abstract

Background: In the geriatric population, the risk of cardiometabolic diseases is strongly influenced by comorbidities. The aim of the study was to estimate the prevalence of depression among hospitalized patients with heart failure (HF) and to assess the relation between physical performance and depression in this population. Methods: We included consecutive hospitalized patients with HF aged >65 years. The depression symptoms were evaluated using the Geriatric Depression Scale (GDS), the physical performance was assessed using the grip strength measurements, the Back Scratch Test, the Timed Up and Go Test (TUGT), the Five Times Sit to Stand Test (5 × SST), and the 6 min walk test. Results: We included 206 patients (134 females and 72 males, median age 82 years (77–86) years). Altogether, 33% of participants had signs of depression. The association was found between depression severity and economic status (*p* = 0.001), stressful events (*p* = 0.005), self-reported general health status (*p* = 0.001), and heart failure severity assessed by the New York Heart Association class (NYHA), *p* = 0.003. The Back Scratch Test, the TUGT, and the 5xSST were associated with depression severity in a univariable regression analysis (β coefficient 0.04 [95% CI 0.00–0.08], 0.20 [95% CI 0.12–0.27], 0.18 [95% CI 0.07–0.27], respectively); however, when adjusted for co-factors, the TUGT and the 5xSST (0.17 [95% CI 0.08–0.26] and 0.14 [95% CI 0.02–0.26], respectively) were significantly related to the GDS score. Grip strength and the 6 min walk test were not related to the GDS score in the univariable nor multivariable analysis. These findings were confirmed in the logistic analyses. Conclusions: Our study indicated a high incidence of depression among elderly hospitalized patients with heart failure. Depression severity in older patients with HF is related to physical performance decline as assessed by the Timed Up and Go Test and the Five Times Sit to Stand Test. Grip strength and the 6 min walk test are not related to the GDS score in this population.

## 1. Introduction

Heart failure (HF) is one of the most frequently diagnosed diseases among older people. It is estimated that heart failure occurs in approximately 6% of people aged 60–79, and this percentage increases significantly in people aged 80 and over [1]. Forecasts indicate an increasing incidence of heart failure in the general population, including persons aged above 60 years. Importantly, more than half of hospitalizations due to heart failure concern the older adult population, mainly people aged 75 years and older [2].

Patients’ health problems may negatively affect psychological and physical aspects of life, thus determining the quality of life of older people. Depression is a common phenomenon among patients with HF. Research shows that depression and anxiety that are comorbid with HF result in increased mortality and more frequent use of health care [3]. Several potential pathways such as endothelial function disturbance, inflammation with pro-inflammatory cytokines (IL1β, IL2, IL6), tumor necrosis factor-α (TNF-α), and C-reactive protein (CRP) increase, but also decline in the autonomic nervous system, and platelet abnormalities are involved as pathophysiological mechanisms connecting depression and cardiovascular diseases (CVDs). It has been reported that in depressive patients with comorbid heart disease, the endothelial dysfunction level is higher in comparison with non-depressive patients with CVD [4]. To confirm this association, studies focused on depressive therapy were conducted. It was highlighted that the selective serotonin reuptake inhibitors (SSRIs) stimulate endothelial function in patients with cardiac and depressive symptoms. Patients diagnosed with depression have also been found to have a significantly higher serum concentration of inflammatory markers. It has been underscored that psychological factors like depressive symptoms can activate stress pathways, resulting in the acceleration of inflammation mechanisms that could lead to the progression of adverse cardiac outcomes in this population of patients among others via atherosclerosis [5].

The overall incidence of depression in this group of patients is approximately 20–30%, and this percentage is even higher in older people [6,7]. Depression is significantly associated with worse outcomes in patients with HF, including poorer patient outcomes and functional decline, resulting in increased hospitalizations and an overall deterioration of health and functioning. Depression is an independent risk factor for cardiac complications and death, as well as a strong predictor of re-hospitalization [8,9].

Importantly, many risk factors for heart failure, such as hypertension and obesity, can be modified [1]. Several studies have indicated that physical activity may reduce depressive symptoms and conversely, low physical activity and performance may be an independent predictor of depression [10,11]. Beyond commonly coexisting, the two diagnoses—depression and heart failure—may magnify one another, with bidirectional effects through both biological as well psychosocial mechanisms.

Considering the above, our study aimed to estimate the prevalence of depression among hospitalized patients with HF and to assess the relation between their physical performance and depression.

## 2. Materials and Methods

### 2.1. Participants and Study Design

The observational prospective study was conducted in the period from June 2022 to January 2024. The study included 206 patients hospitalized and diagnosed with heart failure. Apart from the diagnosed disease (HF as the main cause of hospitalization), the inclusion criteria were the patient’s age above 65 years and their willingness to participate in the study. Patients with severe functional decline, who were not able to perform any physical performance tests, were excluded from the study. In the study group, the grip strength data were available for 183 patients, the Back Scratch Test data were available for 133 patients, the Timed Up and Go Test data were available for 130 patients, the Five Times Sit to Stand Test data were available for 99 patients, and the 6 min walk test data were available for 71 patients.

For each patient, the New York Heart Association class (NYHA) was assessed according to the crucial clinical classification for patients with heart failure, that is, the strongest factor correlating with health status prognosis, allowing us to divide patients into the subgroups according to their functional status.

The research questionnaires included the Geriatric Depression Scale (GDS), standardized forms collecting sociodemographic data, lifestyle, and the health status of patients. As a continuation, after the research questionnaires, the physical performance tests were performed.

### 2.2. Instruments—Questionnaires

The GDS, developed by Yesavage et al., is standardized for elderly patients. The shortened version was used, consisting of 15 questions assessing the occurrence of depressive symptoms in patients (self-assessment method) [12]. Its sensitivity is rated at 92% and specificity at 89%. For each question, patients choose a “yes/no” answer. Each answer is scored 1 or 0 (according to the adopted key). In the GDS, scores range from 0 to 15, with a score of 6/15 points being the threshold for classifying the patient’s symptoms as mild depression, and 10/15 points being classified as major depression cut off points (scores ≥6 indicated mild depression, and scores ≥10 were classified as major depression). The GDS was completed by the patients themselves (paper-and-pencil method), and the remaining information was recorded by researchers using a computer (computer-assisted personal interview). A stressful event in a previous year was defined as an episode of an extremely difficult situation strongly influencing the patients’ lives last year (such as death, an accident, or an experience of violence). Retirement benefits were assessed by the respondent as lower than minimum pension, minimum pension, average, or high. Self-reported physical activity was defined as amount of daily physical activity slots per week (such as a walk, gardening, or shopping). Self-reported general health status was defined as unhealthy, average, and healthy, independent of comorbidities and functional status.

### 2.3. Procedures—Physical Performance Tests

The physical performance of the study participants was assessed when health status was optimized before discharge. It was evaluated using the following tests assessing flexibility and muscle strength and function, using the Fullerton test recommended by the International Council of Sport Science and Physical Education (ICSSPE):

The muscle/grip strength was measured with a dynamometer in a standing position. The tested person bent the arm at the elbow joint at a right angle, squeezing the dynamometer with the hand using maximum force. The measurement was performed twice for each hand and the average measurement was annualized. The conventional unit of grip strength was the kilogram. Dynamometer type FS658 Jamar(JAMAR Technologies, Hatfield, PA, USA) was used.

1. The Back Scratch test assesses upper body flexibility. The test was performed by the patient in a standing position. The patient tried to join both hands behind their back, one reaching from the top to the middle of the back, the other reaching from the bottom. During the test, the missing distance between the middle fingers of both hands was measured in centimeters (to the nearest decimal in centimeters).
2. The Timed Up and Go Test (TUGT) is used to assess the functional mobility of the patient and the fall risk. During the test, the patient used a walking aid that they used in everyday life. From a sitting position, the patient got up, walked 3 m, turned around, and walked 3 m back to the chair. The time in seconds was measured from the start of getting up from the chair to the moment the patient returned to the sitting position. The typically used cut off point for falls is 14 s; for sarcopenia, it is 20 s.
3. The Five Times Sit to Stand Test (5×SST) is used to assess a patient’s ability to transfer from a seated to a standing position and back to sitting five times; the result is based on the amount of time (measured in seconds) needed to perform the test. The typically used cut off point for muscle sarcopenia is 15 s.
4. The 6 min walk test aims to assess the patient’s exercise tolerance. Before starting the test, the patient’s basic vital parameters were measured, including blood pressure, pulse, and blood oxygen saturation. The test measured the distance the patient had walked in six minutes at their own pace. Heart rate and blood oxygen saturation were measured every minute. At the end of the test, blood pressure, pulse, and blood oxygen saturation were reassessed every minute. The typically used cut off point for adults (lack of cut off points for older adults) is 500 m for women and 600 m for men. Blood pressure and blood saturation were measured with automatic devices, a pressure gauge type OMRON M3 COMFORT HEM-7155-E **Omron Corporation**, Kyoto Head Office, Shiokoji Horikawa, Shimogyo ku, Kyoto 600-8530, JAPAN and a finger pulse oximeter type Pro PLSKMR-CTC-007 ADVERTI POLAND respectively.

The study was approved by the Bioethics Committee at the Centre of Postgraduate Medical Education of Warsaw (resolution no. 73/2022 of 8 June 2022), and all study participants gave their informed consent.

### 2.4. Statistical Analysis

Categorical values are presented as percentages, while continuous variables are presented as means (standard deviations [SD]) or medians (interquartile ranges [IQRs]), as appropriate. The Shapiro–Wilk test was used to assess the normality of the continuous variables. The Mann–Whitney U test and the Kruskal–Wallis test were used for variables that had a non-normal distribution. Multivariable analyses were performed on the basis of the generalized linear model, as implemented in the Statistica 13 software (TIBCO Software Inc., Palo Alto, CA, USA). A two-tailed *p*-value of less than 0.05 was considered statistically significant.

The power analysis was conducted to determine the minimum sample size required to test detect the difference in the TUGT between patients with severe depression and those without depression (the proportion of patients with severe depression and the standard deviation of the TUGT results were estimated based on the literature review). The required sample size to achieve 90% power for detecting a 20% difference, at a significance criterion of α = 0.05, was 120.

## 3. Results

The study group consisted of 206 individuals; the median age of the patients was 82 (77–86) years and women constituted 65% of the participants. The majority of the participants received average retirement benefits, while only 3.9% received a very low retirement benefit (Table 1). A similar percentage of participants lived alone or with another person. The predominant type of heart failure was heart failure with preserved ejection fraction (HFpEF). A considerable proportion of the study participants had hypertension, more than 40% of the participants were classified as overweight/obese, and a similar percentage of patients had type 2 diabetes. The participants took between one and twelve medications daily; the median number of medications taken daily was six [5,6,7,8]. The status of weekly performed self-reported physical activity was relatively low (Table 1). All physical performance tests were associated with the NYHA class, while all but the 6 min walk test were associated with age (Table A1). The median GDS score was four (2–7) points. Overall, 138 (67%) study participants had a GDS score below seven points, 46 (22%) between seven and ten points, while 22 (11%) patients had a GDS score of more than 10 points.

We found a significant correlation between the depression severity scale and retirement benefits (*p* = 0.001), the number of stressful events (*p* = 0.005), self-assessed health (*p* = 0.001), and NYHA class status (*p* = 0.003), but not with other assessed variables, including medical and socioeconomic (Table 2).

When the GDS was categorized, the physical performance tests significantly related to the depression signs. These tests were the Back Scratch Test, the Timed Up and Go Test, and the Five Times Sit to Stand Test (Table 3).

When we analyzed the GDS as a continues variable, it appeared to be significantly correlated with the Back Scratch Test, the Timed Up and Go Test, and the Five Times Sit to Stand Test (Table 4). When adjusted for age, sex, the NYHA class, and health status, the Timed Up and Go Test and the Five Times Sit to Stand Test were associated with GDS scoring only. Grip strength and the 6 min walk test were not related to the GDS score in the univariable or multivariable analysis.

Table 5 presents the relations between the physical performance tests and the odds of having severe depression according to the GDS (>10 points). Again, the Back Scratch Test, the Timed Up and Go Test, and the Five Times Sit to Stand Test were associated with a high risk of severe depression in the univariate analysis, while the Timed Up and Go Test and the Five Times Sit to Stand Test remained significantly related to the probability of having severe depression when adjusted for age, sex, NYHA class, and health status, as shown in Table 5. Grip strength and the 6 min walk test were not related to the high GDS score in either the univariable or multivariable analysis.

## 4. Discussion

Our study indicates that about one third of older patients hospitalized due to HF could have comorbid depression, and in the case of over 11% of patients, there were signs of severe depression. Depressive symptoms are influenced by the economic situation of patients (income/pension amount) and stressful events last year that correlate also with self-reported health status.

The Geriatric Depression Scale is considered one of the best tools for assessing depression in older people [2]. Previous studies indicate that the prevalence of severe depressive symptoms in patients with HF is estimated at 36% [9]. Other studies indicate similar results, with around 30% of patients with depression among HF patients and approximately 10% of patients having severe depression [2,7]. However, AbuRuz et al. indicate a higher prevalence of depression among patients with HF, with over 65% [13]. Studies have found higher rates of depression in hospitalized patients with HF (14–77%) compared to outpatients with HF (13–48%), which also coincides with our studies [9].

Research indicates that depression causes the deterioration of physical performance, the severity of HF symptoms, the occurrence of frailty syndrome, poorer quality of life, more frequent use of health care, but also reduced adherence to therapy, which further worsens health effects [6,13,14,15]. Celano et al., examining patients with coronary heart disease and depression, show that these patients are less likely to follow a healthy diet, exercise less often, and do not adhere to pharmacological treatment, which has a significant impact on the lack of expected effects, also in the case of comorbidities. These patients are also less likely to complete cardiac rehabilitation compared to people without depression [8]. Depression, mainly severe, is also an independent predictor of rehospitalization, often multiple. Moreover, it has been proven that depression increases the patient’s risk of death. People with severe depression are four times more likely to die within 2 years compared to patients without depression [9,16]. However, it is still debated if depression treatment reduces the frequency of hospitalizations and to what extent it can improve other treatment outcomes in patients with HF [17]. Depression is therefore considered an independent risk factor for morbidity and mortality due to heart disease. In older people, it is also closely related to cognitive decline and dementia [9]. Despite the high prevalence of depression in older patients with HF and the negative impact of depression on patient outcomes, it is often unrecognized [18]. Our findings highlighted that for the vast majority of participants, it was their first screening for depression and only a few patients were treated because of depression.

Interestingly, all physical performance tests were associated with heart failure severity. It should be noticed that previous studies assessing the physical functioning of patients with HF included a smaller range of physical tests, e.g., single tests such as the 6 min walk test [19]. Age and sex dependence of physical performance highlighted in our study was also highlighted by other researchers [20]. However, there are limited data concerning the group of the oldest population and short physical performance geriatric tools, like the Timed Up and Go Test or the Chair Stand Test, that could be useful in clinical practice.

Physical performance was associated only with chosen medical variables. Atrial fibrillation and myocardial infarction history negatively influenced the results of patients’ physical performance tests. It should be emphasized that in our study, we found a connection between the decline in walking speed and the worsening of depression. Our study also highlighted that comorbidity, including depression and muscle function decline, may play a crucial role in the vicious cycle of risk factors contributing to cardiovascular diseases among the elderly. In everyday clinical practice, short geriatric physical performance tests could become a screening tool for negative outcomes among the elderly with heart failure. It could possibly contribute to secondary prevention, encompassing exercising and nutrition intervention. Studies focused on long-lived individuals showed that low protein serum concentration is the main factor correlated with severe functional decline [21].

Patients with depression are characterized by a deteriorated quality of life, both in terms of physical and mental components, and anxiety coexisting with depression is considered to be independent predictor of poor quality of life [13]. Moreover, the severity of depression is associated with loneliness, the older age of patients, and the severity of the disease, but not all these relationships were confirmed by our study [13,22,23].

The biopsychosocial model of the disease, based on a holistic and multidimensional approach to the care of older patients with HF, indicates that patient functioning and depression are important elements of effective medical therapy. This model covers four areas, including medicine, mind and emotions (including depression), physical performance (mobility assessment), and the social environment [2].

The deterioration of functioning in older patients with HF may be related to the aging process and the development of HF, but also depression. However, depression is an element that is most strongly associated with physical weakness in older people with HF, leading to physical disability [24,25].

Studies indicate significant correlations between the severity of depression and the severity of physical symptoms or deterioration of physical functioning and conversely, studies show a correlation between low levels of physical activity and symptoms of anxiety and depression [9,10].

The influence of physical activity on reducing depressive symptoms in patients with HF has also been proven, where one of the more promising interventions is physical training [26,27,28].

Some studies indicate a relationship between the occurrence of depression and the results of individual fitness tests. For example, in patients with HF in the study by Chialà et al., depression was independently associated with the distance covered in the 6MWT [19]. We did not find similar observations in our study when assessing depression severity; one of the reasons could be the advanced age of our study group. It is worth emphasizing that the 6MWT was indicated as a predictive test for future hospitalizations in patients with HF [29,30]. It is believed that patients with a result of <300 m in the 6MWT are often weaker and have lower exercise capacity [31,32].

Similarly, a comprehensive physical fitness assessment for older women, including the 6MWT, grip strength, 30 s arm curl, 30 s chair stand, the 8 Ft Up and Go Test, the Back Scratch Test, and the Five Times Sit to Stand Test, indicates an association of physical fitness factors with symptoms of depression [33]. This suggests that improving physical fitness may play an important role in preventing depression [11,34].

We showed an independent relationship between the symptoms of depression in older patients and heart failure severity, but not with other cardiovascular diseases including history of stroke, atrial fibrillation, or myocardial infarction. This remains in accordance with the literature data [35,36]. Nevertheless, it is worth emphasizing that reduced exercise capacity and tolerance in patients with HF are associated with the deterioration of patients’ cognitive functions, worse quality of life, and worse prognosis [30]. Most studies clearly indicate that physical activity can support and prevent the symptoms of depression, also in older people [34]. Both aerobic and strength exercises are recommended for this purpose [31].

We found an association between patients’ physical functioning and depression, and we believe that considering both aspects in the assessment of patients with HF may increase awareness in the early identification of people with more advanced HF, which is related to a worse prognosis. Moreover, our study revealed that short physical performance tools, including the TUGT and the 5 × SST, also after adjusting for age, sex, heart failure severity, and self-reported health status, could contribute to more multifactorial diagnostic process as they are significantly associated with the severity of depression in elderly patients. According to the literature data, after adjusting for relevant variables, patients with depression and heart failure have reported poorer cognitive and physical performance. Researchers have also underlined that patients with heart failure and more advanced functional decline experience greater depressive clinical symptoms [37,38]. Lastly, a published meta-analysis highlighted that cognitive behavioral therapy may be more effective than standard therapy at ameliorating depression scores and quality of life in older patients with heart failure, which is in concordance with the effectiveness of multifactorial interventions in the geriatric population [39].

Late-life depression can present subclinical depressive symptoms coexisting with reduced stress tolerance. If unchecked, it can progress, especially in patients with heart disease. Various biological and environmental factors across the lifespan including inflammation, muscle mass loss, and strength decline increase vulnerability and become crucial for pathways of this disequilibrium. In turn, depressive episodes alter nutrition intake that accelerate sarcopenia and contribute to adverse long-term outcomes such as an increase in cardiovascular events [40]. Our study underscored the role of comorbidities in the elderly; in this population, not only cardiovascular diseases but also typical geriatric syndromes like depression contribute to its clinical impact and the acceleration of mobility decline. Therefore, coexistence of these three processes (CVD, depression, and sarcopenia symptoms) and their role in the vicious cycle should be considered. These mechanisms strongly contribute to physical decline and may play a crucial role in everyday clinical practice and patients’ quality of life deterioration. Moreover, in everyday clinical practice, short physical performance tests could become complementary to NYHA classification in the assessment of the elderly.

Further studies of hospitalized patients with HF are necessary to determine the extent to which the physical functioning of patients correlates with the occurrence of depressive symptoms and the role of comorbidities and its impact on physical performance, as well as pathophysiological mechanisms of the process including inflammation, endothelium decline, and gender issues.

## 5. Study Limitations

The present study has several limitations. Firstly, this is a single-center study with a limited number of cases. Secondly, the evidence suggests depression increases the risk of hospitalization. Since we studied hospitalized patients, our group may have included more people with symptoms of depression than in the general sample of older people with HF. Indeed, as the study participants were hospitalized, we studied patients aged >65 years; the present results do not refer directly to non-hospitalized and younger patients. Moreover, in our study, we found that only a limited number of patients proceeded with subsequent physical tests and it might be more relevant for real clinical practice, as elderly patients would not be able to perform the subsequent tests or feel tired with the lengthy procedure of examinations. However, an important advantage of our analysis is that our results are not based solely on abstracted medical record data but considered face-to-face interviews and examinations using the same protocol and standardized methods and instruments. Therefore, to the best of our knowledge, the presented results provide the most reliable information on the relation between depression and physical performance in older patients hospitalized for heart failure.

## 6. Conclusions

Our study indicated a high incidence of depression among elderly hospitalized patients with heart failure. Depression severity in older patients with HF is related to physical performance decline, as assessed by the Timed Up and Go Test and the Five Times Sit to Stand Test. Grip strength and the 6 min walk test are not related to the GDS score in this population.

## Figures and Tables

**Table 1:** Characteristics of the analyzed group. *N* = 206.

Variable	Median (1st–3rd Quartile) or Number (%)
Age, years	82 (77–86)
Sex	
Females	134 (65.0)
Males	72 (35.0)
Socio-economic indicators	
Retirement benefits	
Very low	8 (3.9)
Low	45 (21.8)
Average	128 (62.1)
High	25 (12.2)
Accommodation	
Alone	100 (48.6)
With company	104 (50.5)
Nursing home	2 (0.9)
Stressful events last year	
0	113 (54.5)
1	54 (26.3)
2	24 (11.6)
3	12 (5.8)
4	4 (1.8)
Geriatric Depression Scale	
Without depression	138 (67.0)
Mild depression	46 (22.0)
Severe depression	22 (11.0)
Physical performance indicators **	
Grip strength right hand, kg	20.05 (15.95–28.50)
Grip strength left hand, kg	18.70 (13.35–26.40)
Grip strength average, kg	19.20 (14.25–28.0)
Back Scratch Test, cm	15.0 (6.0–26.0)
Timed Up and Go Test, s	13.33 (9.82–19.13)
Five Times Sit to Stand Test, s	17.36 (13.57–21.35)
6 min walk test, m	305.0 (210.0–360.0)
Self-reported physical activity, times per week	
0	81 (39.3)
1–3	42 (20.4)
>3	83 (40.3)
Medical variables	
Self-assessment of general health status	
Unhealthy	27 (13.0)
Average	98 (47.7)
Healthy	81 (39.3)
Number of medication intake	
<5	52 (25.3)
≥5	154 (74.7)
Obesity	90 (43.7)
Smoking	91 (44.2)
NYHA * class	
I	94 (45.6)
II	50 (24.3)
III	48 (23.3)
IV	14 (6.8)
Left ventricular ejection fraction, %	50.0 (50.0–66.0)
Prior myocardial infraction	31 (15.1)
Atrial fibrillation	78 (37.7)
Hypertension	171 (83.0)
Prior stroke	24 (11.6)
Diabetes	90 (43.7)
Chronic renal failure	62 (30.0)

* NYHA—New York Heart Association. ** Grip strength—data available for 183 patients. Back Scratch Test—data available for 133 patients. Timed Up and Go Test—data available for 130 patients. Five Times Sit to Stand Test—data available for 99 patients. The 6 min walk test—data available for 71 patients.

**Table 2:** Association between Geriatric Depression Scale and socioeconomic and clinical variables.

	Geriatric Depression Scale			*p*-Value
	Without Depression	Mild Depression	Severe Depression	
Age, years	82.0 (77–85)	81.0 (77–86)	84.5 (79–89)	0.423
Sex, male	46 (33.33%)	19 (41.30%)	7 (31.80%)	0.585
Sex, female	92 (66.67%)	27 (58.70%)	15 (68.20%)	
Socioeconomic variables				
Very low and low retirement benefits	23 (16.67%)	19 (41.30%)	11 (50.00%)	0.001
Average retirement benefits	95 (68.84%)	23 (50.00%)	10 (45.45%)	
High retirement benefits	20 (14.49%)	4 (8.70%)	1 (4.55%)	
Stressful events last year ≥2	91	57	43	0.005
Accommodation				
Alone	68	21	11	0.883
With company	69	25	10	
Nursing home	1	0	1	
Medical variables				
Self-reported general health status (healthy or average)	128	36	15	0.001
Drugs ≥ 5	100 (72.5%)	37 (80.4%)	17 (77.3%)	0.314
Smoking	66 (47.83%)	17 (36.96%)	8 (36.36%)	0.322
Obesity	58 (42.03%)	21 (45.65%)	11 (50.00%)	0.747
NYHA * class I, II	105	30	9	0.003
NYHA class III, IV	33	16	13	
Atrial fibrillation	52 (37.68%)	17 (36.96%)	9 (40.91%)	0.948
Prior myocardial infarction	19 (13.77%)	8 (17.93%)	4 (18.18%)	0.762
Prior stroke	18 (13.04%)	2 (4.35%)	4 (18.18%)	0.169
Hypertension	115 (83.33%)	40 (86.96%)	16 (72.73%)	0.338

* NYHA—New York Heart Association.

**Table 3:** Relation of depression severity and physical performance tests.

Variables	Geriatric Depression Scale			*p* Value
	Without Depression *N* = 138	Mild Depression *N* = 46	Severe Depression *N* = 22	
Grip strength, kg	19.22 (15.15–27.32)	19.26 (15.78–29.44)	16.77 (8.90–28.63)	0.998
Back Scratch Test, cm	15.0 (6.0–26.0)	14.0 (3.0–22.0)	33.0 (24.0–34.0)	0.044
Timed Up and Go Test, s	12.89 (9.26–18.0)	12.83 (10.25–16.80)	21.50 (18.30–31.50)	0.002
Five Times Sit to Stand Test, s	16.64 (13.0–20.85)	16.89 (14.78–22.93)	22.00 (21.20–28.0)	0.021
6 min walk test, m	305.00 (204.8–362.1)	303.50 (202.5–347.5)	202.0 (120.0–252.0)	0.216

**Table 4:** The relation between physical performance and Geriatric Depression Scale scoring in multivariate regression analysis.

Variable	Univariate Analysis	Multivariate Analysis		
		Adjusted for Age and Sex	Adjusted for Age, Sex, and NYHA Class	Adjusted for Age, Sex, NYHA Class, and Health Status
	β Coefficient (95% CI)			
Grip strength	−0.03 (−0.02–0.08)	−0.03 (−0.02–0.09)	−0.02 (−0.08–0.04)	−0.02 (−0.08–0.04)
The Back Scratch Test	0.04 (0.00–0.08)	0.04 (0.00–0.08)	0.03 (−0.01–0.07)	0.03 (−0.01–0.06)
The Timed Up and Go Test	0.20 (0.12–0.27)	0.19 (0.10–0.27)	0.15 (0.06–0.24)	0.17 (0.08–0.26)
Five Times Sit to Stand Test	0.18 (0.07–0.27)	0.17 (0.06–0.28)	0.15 (0.03–0.27)	0.14 (0.02–0.26)
The 6 min walk test	−0.06 (−0.13–0.01)	−0.05 (−0.12–0.02)	−0.01 (−0.08–0.06)	−0.01 (−0.08–0.06)

NYHA—New York Heart Association.

**Table 5:** The relation between physical performance and severe depression in multivariate logistic analysis (Geriatric Depression Scale, GDS > 10).

Variable	Univariate Analysis	Multivariate Analysis		
		Adjusted for Age and Sex	Adjusted for Age, Sex, and NYHA Class	Adjusted for Age, Sex, NYHA Class, and Health Status
	Odds Ratio (95% CI)			
Grip strength	0.96 (0.91–1.01)	0.94 (0.87–1.01)	0.95 (0.88–1.02)	0.95 (0.88–1.02)
The Back Scratch Test	1.05 (1.01–1.10)	1.05 (1.00–1.10)	1.04 (1.00–1.10)	1.04 (0.99–1.10)
The Timed Up and Go Test	1.17 (1.07–1.27)	1.13 (1.04–1.24)	1.13 (1.02–1.25)	1.19 (1.05–1.36)
Five Times Sit to Stand Test	1.19 (1.04–1.36)	1.22 (1.03–1.45)	1.25 (1.00–1.50)	1.22 (1.00–1.50)
The 6 min walk test	0.92 (0.81–1.03)	0.92 (0.79–1.07)	0.94 (0.79–1.11)	0.94 (0.79–1.11)

## Data Availability

Data are contained within the article.

## List of Abbreviations

AF	atrial fibrillation
CAD	coronary artery disease
CAPI	computer-assisted personal interviewing
COPD	chronic obstructive pulmonary disease
GDS	Geriatric Depression Scale
HF	heart failure
HFmEF	heart failure with mid-range ejection fraction
HFpEF	heart failure with preserved ejection fraction
HFrEF	heart failure with reduced ejection fraction
ICSSPE	International Council of Sport Science and Physical Education
NYHA	New York Heart Association
SD	standard deviation
6MWT	6 min walk test

## Appendix A

**Table A1:** The association between physical performance tests, and socio-economic status, and clinical indica-tors.

Variables	Grip Strength, kg	Back Scratch Test, cm	Timed Up and Go Test, s	5x Sit-To-Stand Test, s	6-min Walk Test, m
	Median [1st–3rd Quartile]				
Age, ≤82 years	22.01 [15.90–31.08]	13.00 [4.00–23.00]	11.66 [9.00–16.10]	15.09 [12.39–19.19]	324.00 [243.00–377.00]
Age, >82 years	18.20 [11.65–21.43]	20.00 [10.00–33.00]	16.25 [11.95–21.50]	19.40 [15.80–24.90]	252.00 [202.00–315.00]
*p* value	0.001	0.007	0.001	0.001	0.153
Sex, female	16.84 [11.65–20.88]	15.0 [5.0–27.0]	15.05 [11.66–19.70]	18.58 [13.76–22.0]	303.0 [202.0–337.0]
male	28.45 [21.85–34.25]	18.00 [9.00–25.50]	10.28 [8.40–14.16]	16.40 [17.70–19.27]	326.0 [238.0–405.0]
*p* value	0.000	0.251	0.0001	0.149	0.032
Retirement benefits					
Very low/low	17.98 [14.28–26.75]	15.00 [4.00–25.00]	14.90 [10.46–19.46]	18.29 [14.35–21.70]	282.00 [210.00–312.00]
Average	18.33 [13.75–26.25]	16.00 [5.00–27.00]	13.56 [9.56–19.11]	18.36 [13.61–22.14]	305.00 [202.00–360.00]
High	22.65 [18.25–33.80]	17.50 [13.00–32.00]	13.72 [10.30–19.30]	14.69 [12.88–16.53]	300.00 [210.00–326.00]
*p* value	0.094	0.332	0.650	0.353	0.633
Smoking history	20.95 [16.40–29.00]	16.00 [5.00–26.00]	12.71 [9.82–17.12]	17.29 [13.57–21.20]	303.00 [201.00–356.00]
No smoking history	18.34 [12.68–25.88]	14.50 [7.00–27.00]	14.81 [9.81–19.90]	17.94 [13.60–21.35]	305.00 [238.00–380.00]
*p* value	0.019	0.916	0.121	0.813	0.909
Overweight/obesity	19.20 [13.75–28.45]	15.00 [4.00–25.00]	13.89 [10.00–18.30]	16.99 [12.98–21.50]	301.00 [208.00–364.00]
Without overweight/obesity	19.29 [15.57–27.25]	16.00 [8.00–29.00]	13.00 [9.62–20.00]	17.82 [14.05–21.35]	305.0 [238.0–360.0]
*p* value	0.582	0.244	0.887	0.745	0.599
NYHA I*	20.80 [15.97–29.49]	15.00 [4.50–23.0]	12.20 [9.26–16.00]	16.11 [12.10–19.0]	325.0 [296.0–380.0]
NYHA II	19.05 [15.68–25.65]	10.50 [5.0–23.0]	12.00 [9.30–16.50]	16.8 [13.81–23.14]	259.0 [208.0–360.0]
NYHA III	19.21 [10.99–29.48]	22.00 [13.00–44.00]	18. 50 [13.91–23.25]	21.70 [17.30–26.30]	202.0 [163.0–252.0]
NYHA IV	14.85 [10.43–17.98]	14.00 [10.00–21.00]	23.10 [20.40–33.55]	19.40 [19.40–19.40]	195.00 [195.00–195.00]
*p* value	0.046	0.021	0.0004	0.014	0.046
Atrial fibrillation	17.25 [11.84–25.06]	22.00 [12.00–36.00]	15.86 [10.00–29.60]	18.48 [14.75–21.30]	301.00 [238.00–356.00]
No atrial fibrillation	20.88 [15.60–29.03]	12.50 [4.00–22.00]	12.92 [9.64–18.00]	16.86 [12.40–21.35]	305.00 [208.00–375.00]
*p* value	0.014	0.001	0.197	0.192	0.850
Previous stroke	16.53 [11.30–20.28]	15.50 [9.0–24.0]	18.37 [14.00–19.60]	21.04 [17.89–24.35]	231.0 [182.0–286.0]
No previous stroke	19.83 [15.15–28.45]	15.0 [5.0–27.0]	12.98 [9.66–19.06]	17.29 [13.55–21.30]	306.0 [225.0–364.0]
*p* value	0.111	0.887	0.094	0.089	0.125
Previous myocardial infarction	18.03 [11.48–29.74]	21.00 [7.0–40.50]	13.43 [11.92–18.37]	17.30 [14.75–21.0]	199.0 [105.0–293.0]
No previous myocardial infarction	19.60 [14.85–27.58]	15.00 [6.0–35.0]	13.33 [9.64–19.38]	17.60 [13.55–21.70]	306.0 [233.0–375.0]
*p* value	0.564	0.092	0.859	0.958	0.011

* NYHA—New York Heart Association.
